# Randomized Trial on Electroacupuncture for Recovery of Postoperative Gastrointestinal Function Based on Long-Term Monitoring Device

**DOI:** 10.1245/s10434-025-17239-3

**Published:** 2025-04-04

**Authors:** Yuling Cai, Haoyang Li, Haiou Nan, Pengyan Xu, Jiayu Li, Huafeng Pan, Haifeng Wang, Miaomiao Ge, Junjie Guan, Zhiwei Jiang, Gang Wang

**Affiliations:** 1 First Clinical Medical College, Nanjing University of Chinese Medicine, Nanjing, Jiangsu China; 2 Affiliated Hospital of Nanjing University of Chinese Medicine, Nanjing, Jiangsu China

**Keywords:** Gastric cancer, Postoperative gastric dysfunction, Electroacupuncture, Gastrointestinal functional recovery, HRV, Long-term HRV monitoring, POI

## Abstract

**Background:**

This research aimed to explore the efficacy and safety of electroacupuncture in promoting the recovery of postoperative gastrointestinal function and to discuss the potential mechanism on the basis of heart rate variability (HRV).

**Patients and Methods:**

This was a randomized controlled study. Postoperative patients with gastric cancer received either electroacupuncture (EA) or sham electroacupuncture (SEA) 2 h after surgery and on the morning of the first 2 days after surgery, with each session lasting 30 min. The acupoints, treatment timepoints, and treatment durations in the SEA group were kept consistent with those in the EA group, but the intervention was SEA. Both groups were equipped with artificial intelligence HRV monitoring devices to monitor perioperative HRV and continuous bowel sound auscultation recorders to monitor perioperative bowel sound recovery in real time. Gastrointestinal function recovery indicators, HRV indicators, inflammatory markers, the incidence of postoperative complications, and adverse events were analyzed.

**Results:**

There was no statistically significant difference (*P* > 0.05) in baseline. First flatus time, first oral feeding time, intestinal function recovery time, and length of postoperative hospitalization of EA group were better than those of the SEA group, (*P* < 0.05). On day 3 after surgery, in EA group, C-reactive protein, interleukin-1 beta (IL-1*β*) were lower than those in SEA group (*P* < 0.05). HRV indicators such as standard deviation of the average NN intervals, percentage of successive RR intervals that differ by more than 50 ms (PNN50), and high frequency were higher in EA group than those in SEA group (*P* < 0.05).

**Conclusions:**

EA can safely and effectively promote gastrointestinal function rehabilitation in postoperative patients with gastric cancer, whose mechanism may be associated with higher tension in the vagus nerve, affected by EA.

Gastric cancer is one of the most common malignant tumors, whose incidence increases annually, and currently, the most effective treatment is surgery.^1^ Postoperative ileus (POI) frequently occurs in postoperative patients with gastric cancer^2^ with characteristic gastrointestinal dysfunction symptoms such as nausea and vomiting, abdominal distension and pain, inability to tolerate diet, difficulty in defecation, and weakened or absent bowel sounds. The occurrence of POI results from the synergistic effects of multiple factors, including autonomic nervous system disorders, gastrointestinal stress, inflammatory reactions, and pharmacological effects. Studies^3^ indicate that risk factors include history of abdominal surgery, type and duration of gastric surgery, degree of abdominal adhesion, postoperative history of opioid use, tumor, node, metastasis (TNM) period of the tumor, and postoperative blood transfusion. Since the continuous progress of POI prolongs patients’ hospitalization time, increases hospitalization costs, and even affects prognosis, accelerating the recovery of postoperative gastrointestinal function is a focus of attention after gastric cancer surgery.

There have been no clear prevention and treatment measures for POI yet. Pharmacological therapy and nonpharmacological therapy are two main methods in present clinical practice. Although pharmacological therapy has been demonstrated to be effective, the incidence of adverse reactions can be relatively high. Nonpharmacological treatments, including chewing gum, minimally invasive surgery, and enhanced recovery after surgery (ERAS) measures, have only limited effectiveness in preventing and treating POI. At present, the research on prevention and control of POI has reached a bottleneck. Hence, a complementary therapy, which can prove to have high potential for suppressing the development of POI, is urgently needed. Acupuncture therapy has been applied in China for thousands of years and has been receiving increasing attention worldwide over the past few decades. Acupuncture has the advantage of bidirectional regulation, exerting a regulatory effect on gastrointestinal motility through a complex neuro-endocrine-immune network.^4^ It also has the merits of fewer adverse side effects, high safety, and easy operation. Acupuncture, especially EA, can shorten flatus, defecation time, and hospitalization time of patients with POI after abdominal surgery and promote the recovery of gastrointestinal function.^5^ Therefore, EA can be an essential method to prevent the occurrence and contain the progress of POI.

The autonomic nervous system is composed of the sympathetic nervous system and the parasympathetic nervous system (vagus nerve), which plays an important role through mutual antagonism in regulating gastrointestinal movement, maintaining dynamic balance between the internal and external environment, and servicing normal life activities. When gastrointestinal dysfunction occurs, it can be observed that vagus nerve activity is restricted and autonomic nervous system is dysfunctional.^6^ Therefore, the mechanism of EA treatment for POI may be related to the upregulation of vagus nerve activity. This study applied artificial intelligence monitoring devices (wearable dynamic electrocardiogram recorder and continuous bowel sound auscultation recorder) to clinically observe the effect of EA on postoperative intestinal function recovery in postoperative patients. Through long-term analysis of dynamic, visual, and digital multi-data, the effectiveness and safety of EA for POI was clarified.

## Patients and Methods

This is a single-center, two-arm, parallel, single blind, randomized controlled trial. It was designed in accordance with the Declaration of Helsinki and the ethical principles of the International Conference on Harmonization-Good Clinical Practice. The ethics protocol was approved by the Medical Ethics Committee of the Affiliated Hospital of Nanjing University of Chinese Medicine (2021NL-053-03). Written informed consent was signed by the patients and the researchers. Data were collected, analyzed, and reported according to the Consolidated Standards of Reporting Trials (CONSORT) statement.^7^ The trial was retrospectively registered at Clinicaltrials.gov (ChiCTR2100050660). No important change was made during the implementation stage. If there had been a clear difference in the effects of different interventions in the early stage of the trial, the trial would have been terminated early for ethical reasons.

### Inclusion Criteria

Patients with gastric cancer who received laparoscopic radical resection and who were admitted to the Department of General Surgery of Affiliated Hospital of Nanjing University of Chinese Medicine were selected from September 2022 to June 2024. There was no gender limitation.

The inclusion criteria were as follows: (1) age older than 18 years old; (2) clinical diagnosis of gastric cancer based on medical history, endoscopic imaging examination, and pathological results; (3) patient agreed to and received the radical gastrectomy for gastric cancer; (4) American Society of Anesthesiologists (ASA) fitness grade of I–III; (5) patients able to comply with protocol during study period, voluntarily participate in the trial, and sign an informed consent form; (6) proximal, distal, and complete gastrectomy carried out with Giraffe reconstruction, Billroth II reconstruction, and Roux-en-Y reconstruction, respectively.

**Fig. 1:**
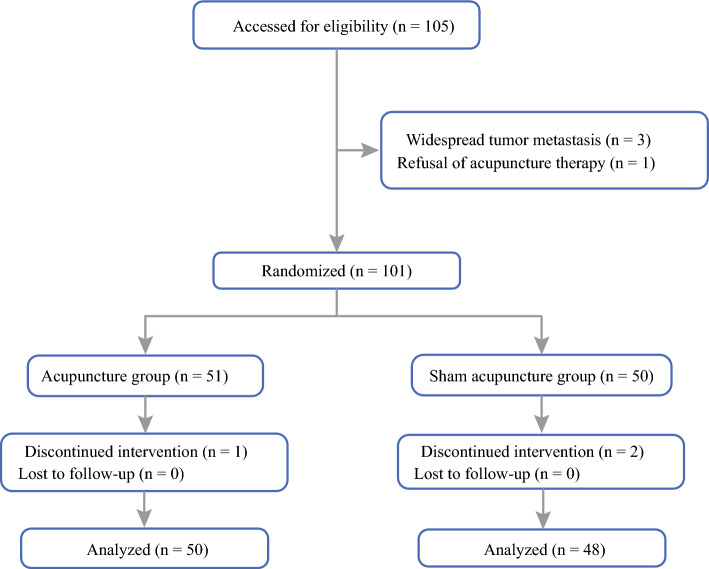
Flow diagram of the study

Patients were excluded if they had any of the following conditions: (1) widespread abdominal metastasis or distant metastases from other organs and tissues; (2) history of abdominal surgery; (3) serious complications or severe infections after surgery; (4) skin damage or infection in the acupuncture area; (5) implantation of electrophysiological devices in the body; (6) had received acupuncture or massage treatment within the 6 months prior to the start of the study; (7) were allergic to patches due to dizziness or allergies (Fig. 1).

### Randomization and Blinding

The random number sequence was generated by an independent statistician who did not participate in any other part of the study. The sequential number was sealed into opaque envelopes and kept by the project coordinator. Patients were then randomly allocated into acupuncture or sham acupuncture group in a ratio of 1:1 by block randomization. Acupuncturists could not be blinded owing to the acupuncture procedure. The patients had an eye patch placed over their eyes to achieve blinding. The assessors of outcome measures and the statisticians performing the data analysis were blinded.

### Interventions

The Enhanced Recovery After Surgery (ERAS) clinical pathway was adopted and the specific management referred to *Clinical practice guidelines for enhanced recovery after surgery in China* (*2021 edition*).^8^ The ERAS treatment methods for gastric cancer mainly include preoperative preparation, intraoperative management, and postoperative management, and were as follows: (1) patients without gastrointestinal motility disorders should fast for 6 h and be prohibited from drinking for 2 h before surgery; (2) minimum dose anesthesia and multimodal analgesia; (3) unconventional use of nasogastric tubes and unconventional placement of abdominal drainage tubes after surgery; (4) early postoperative activity and eating. All patients received standardized ERAS treatment, in which there was no difference in narcotic use, pain medication, and pro-motility agents.

EA group: bilateral ST36 Zusanli, bilateral SP06 Sanyinjiao, and bilateral LI4 Hegu were selected. These three acupoints can relieve postoperative abdominal distension, abdominal pain, diarrhea, and constipation. A disposable sterile acupuncture needle (0.30 mm × 40 mm, Suzhou Hwatuo Medical Equipment Co., Ltd.) was inserted along the anatomical location, directly piercing 2–4 cm. Each insertion was required to make the patient feel obvious soreness, numbness, or swelling spreading to the ankle. The electric acupuncture device (Hwatuo, SDZ-V) was connected to a needle handle. The positive and negative poles were respectively connected to the ST36 Zusanli and SP06 Sanyinjiao on the same side using a continuous wave with a frequency of 10 Hz and an intensity of 2–5 mA. The current was adjusted to the patient’s tolerable intensity range. The needles were left for 30 min.

SEA group: the operations were the same as the EA group, except that there was no current after the needle was connected to the electrode.

Bowel sound monitoring devices: a bowel sound acquisition patch, bowel sound receiver, near field communication (NFC) reader/writer, and supporting software (YM-TYJL-01, Shandong Yimai Medical Technology Co., Ltd., production batch no. TPD181202) were applied to dynamically monitor the bowel sounds and display the number of bowel sounds per min and the cumulative occurrence time of bowel sounds per min. Patch was activated and patient information was inputted. Then, after the patient returned to the ward after surgery, the patch was placed near the McPherson point. The patch collected raw bowel sound data and wirelessly transmitted these data to the terminal for intelligent processing. This device has been proven to be accurate and effective in early clinical applications.^9^

HRV monitoring devices: a wearable dynamic electrocardiograph (TES010, SoSi Medical Technology Co., Ltd., Suzhou, China) was applied to implement dynamic electrocardiogram monitoring. After the patient was admitted to the hospital, the researchers cleaned the skin in the precordial area with alcohol swabs and physiological saline and then attached the registered smart device to the left chest. Then the heart rate and HRV were monitored throughout the entire process from 1 day before surgery to 4 days after surgery. After the patient was discharged, the data from the wearable dynamic electrocardiogram recorder were inputted into the terminal for intelligent processing. The indicators included frequency domain analysis indicators: low frequency (LF), high frequency (HF), low frequency/high frequency (LF/HF); time domain analysis indicators: percentage of difference between adjacent normal R-R intervals exceeding 50 ms (pNN50) and standard deviation of NN intervals (SDNN), standard deviation of the average NN intervals (SDANN) and SDNNIndex.^10,11^ LF reflects the combined tone of the sympathetic and vagus nervous systems. It gives an indication of the overall balance between these two key components of the autonomic nervous system. HF reflects the tone of the parasympathetic nervous system. Higher HF value indicates a stronger parasympathetic tone. SDNN, SDANN and SDNNIndex reflects the overall tone of the autonomic nervous system. A larger SDNN, SDANN or SDNNIndex value implies a more active and balanced autonomic regulation. PNN50 reflects the vagal tone. Higher pNN50 value is associated with increased parasympathetic influence.

### Outcomes

The primary outcome was first flatus time. Secondary outcomes included first oral feeding time, bowel sound recovery time and length of hospitalization after surgery. Serum inflammatory cytokine indicators on day 1 and day 3 after surgery included: C-reactive protein (CRP), white blood cell (WBC) count, interleukin-1 beta (IL-1β), interleukin-2 (IL-2), interleukin-6 (IL-6), tumor necrosis factor-α (TNF-α), and interferon-α (IFN-α). HRV indicators on postoperative day 1 and 3 included: SDNN, pNN50, LF, HF, and LF/HF. Occurrence of dizziness, needle breakage, needle retention, local hematoma, allergies, infections, and other discomforts after acupuncture in patients was observed and recorded, and symptomatic treatment was provided. The Clavien–Dindo classification system^12^ was utilized to record postoperative complications and compare the incidence of postoperative complications between the two groups and analyze the safety of EA.

### Statistical Analysis

The sample size was determined by how many patients met the inclusion criteria without an upper limitation. SPSS 26.0 was used for data statistical analysis. Normal distribution measurement data was expressed as mean ± standard deviation (X̄ ± s) and an independent sample *t*-test was used for intergroup comparison. Non-normal distribution metric data are represented by median and interquartile range (M(IQR)). The Wilcoxon rank sum test was used for intergroup comparison. Count data were presented as examples and the *χ*^*2*^ test or Fisher’s exact probability method was used to perform the group-to-group comparison.

## Results

### Patient Characteristics

In total, 105 patients met the inclusion criteria. Of them, seven were excluded because of widespread tumor metastasis (*n* = 3), refusal of acupuncture therapy (*n* = 1), and incomplete treatment (*n* = 3). However, 98 patients completed the treatment and were enrolled in the statistical analysis. The baseline features of the two groups are presented in Table 1. There was no statistical difference in baseline characteristics between the groups (*P* > 0.05).

**Table 1:** Baseline features of the patients in two groups

Variable	EA	SEA	*T*/*χ*^2^/*Z*-value	*P*-value
*Age (years)*	64.0 ± 9.8	64.2 ± 11.0	−0.07	0.944
*Gender*			0.331	0.565
Male	32 (64%)	28 (58.3%)		
Female	18 (36%)	20 (41.7%)		
*BMI (kg/m*^*2*^*)*	23.7 ± 3.3	23.9 ± 2.8	−0.203	0.84
*ASA fitness degree* (*n*)			0.328	0.567
I	0 (0%)	0 (0%)		
II	36 (72%)	32 (66.7%)		
III	14(28%)	16 (33.3%)		
*Primary procedure*				
Proximal stomach	8 (16%)	8 (16.7%)	0.238	0.888
Distal stomach	23 (46%)	24 (50%)		
Whole stomach	19 (38%)	16 (33.3%)		
*TNM classification* (*n*)			0.981	0.612
I	16 (32%)	13 (27.1%)		
II	17 (34%)	14 (29.2%)		
III	17 (34%)	21 (43.8%)		
*Lymph node metastasis* (*n*)			1.963	0.161
Yes	31 (62%)	23 (47.9%)		
No	19 (38%)	25 (52.1%)		
*Surgical time (min)*	259.7 ± 53.2	278.0 ± 43.8	−1.852	0.067
*Bleeding volume (mL)*	50 (30–50)	50 (40–60)	−1.412	0.158
*Preoperative levels of inflammatory factors*				
CRP/(mg/L)	0.5 (0.5–1.6)	0.5 (0.5–1.5)	−0.141	0.888
WCB/(10^9^/L)	4.9 (4.5–6.0)	4.7 (4.1–5.7)	−0.892	0.372
IL-1β/pg/mL	6.5 (3.3–12.9)	5.3 (1.9–12.3)	−0.59	0.555
IL-2/pg/mL	2.2 (1.7–2.7)	2.0 (1.4–2.5)	−0.896	0.37
IL-6/pg/mL	3.8 (2.6–6.8)	2.6 (1.8–4.7)	−1.944	0.052
TNF-α/pg/mL	1.7 (1.0–3.6)	1.3 (0.7–2.2)	−1.723	0.085
IFN-γ/pg/mL	6.3 (3.7–11.0)	4.0 (2.2–7.6)	−1.734	0.083
*Preoperative HRV indicators*				
SDNN/ms	101.4 (86.4–121.9)	99.0 (72.0–140.0)	−0.071	0.943
SDNNIndex/ms	48.2 (35.4–54.5)	46.2 (33.8–57.5)	−0.06	0.952
SDANN/ms	60.1 (43.8–88.9)	61.6 (34.1–80.9)	−0.718	0.473
PNN50/%	3.0 (1.0–10.6)	3.7 (1.2–11.0)	−0.22	0.826
LF/ms	401.4 (251.8–646.1)	372.6 (263.0–714.6)	−0.235	0.815
HF/ms^2^	254.8 (127.6–570.5)	294.0 (123.1–418.6)	−0.206	0.837

Data are presented in the form of mean ± standard deviation (X̄ ± s) or median and interquartile range (M(IQR)) or percentage (%)

There was no statistically significant difference in age, gender, surgical method, tumor size, TNM stage, lymph node metastasis, surgical time, bleeding volume, and preoperative levels of inflammatory factors in the two groups (*P* > 0.05) after statistical assessment.

### Comparison of Indicators for Intestinal Function Recovery

The first flatus time, first oral feeding time, bowel sound recovery time, and length of postoperative hospitalization in the EA group were shorter than those in the SEA group, and the differences were statistically significant (*P* < 0.05), as presented in Table 2.

**Table 2:** Comparison of indicators for intestinal function recovery

Indicators	EA	SEA	*T*-value	*P*-value
First flatus time (h)	42.7 (31.7–48.2)	46.8 (43.7–64.6)	−3.28	0.001^*^
First oral feeding time (h)	62.2 (42.3–91.3)	68.3 (62.0–131.8)	−2.37	0.018^*^
Bowel sound recovery time (h)	34.5 (26.3–41.6)	40.7 (38.4–44.8)	−3.827	< 0.001^*^
Length of postoperative hospitalization (day)	5 (4–6)	5 (5–6)	−2.138	0.033^*^

Compared with the SEA group, * *P* < 0.05. Data are presented in the form of median and interquartile range (M(IQR))

### Comparison of HRV Indicators

The SDANN, PNN50, and HF in the EA group were higher than those in the SEA group and the differences were statistically significant (*P* < 0.05), as presented in Table 3.

**Table 3:** Comparison of HRV indicators

HRV indicators	EA	SEA	*T*-value/*Z*-value	*P*- value
First day after surgery				
SDNN/ms	98.4 (68.7–150.5)	85.6 (55.9–137.9)	−1.421	0.155
SDNNIndex/ms	49.7 (36.9–71.8)	42.5 (32.4–53.6)	−1.912	0.056
SDANN/ms	65.1 (45.3–104.3)	70.5 (50.0–94.3)	−0.199	0.842
PNN50/%	6.3 (2.2–19.9)	4.4 (1.3–10.7)	−1.066	0.286
LF/ms^2^	665.5 (356.9–1535.5)	481.27 (280.2–752.5)	−1.933	0.053
HF/ms^2^	513.8 (212.5–1611.6)	387.0 (209.5–788.1)	−1.713	0.087
Third day after surgery				
SDNN/ms	86.7 (68.3–137.6)	91.7 (79.4–123.9)	−0.775	0.439
SDNNIndex/ms	48.6 (39.1–67.6)	43.9 (36.5–66.2)	−0.672	0.502
SDANN/ms	98.9 (78.7–126.8)	85.0 (61.0–111.7)	−2.011	0.044^*^
PNN50/%	12.4 (4.8–19.3)	6.2 (2.4–18.2)	−2.118	0.034^*^
LF/ms^2^	536.0 (357.3–1473.4)	526.8 (344.8–963.0)	−0.142	0.887
HF/ms^2^	973.8 (333.8–2337.5)	510.6 (271.0–1066.7)	−2.033	0.042^*^

Compared with the SEA group, * *P* < 0.05. Data are presented in the form of median and interquartile range (M(IQR))

### Comparison of Postoperative Inflammatory Markers

On day 1 after surgery, there was no statistical difference in CRP, WCB, IL-1*β*, IL-2, IL-6, TNF-*α*, IFN-*γ*, and other indicators between the two groups. On day 3 after surgery, the levels of CRP and IL-1*β* in the EA group were lower than those in the SEA group and the level of IL-6 was higher in the EA group than that in the SEA group. The statistical differences (*P* < 0.05) are presented in Table 4.

**Table 4:** Comparison of postoperative inflammatory markers

Inflammatory markers	EA	SEA	*T*-value/*Z*-value	*P*-value
*First day after surgery*				
CRP/(mg/L)	42.2 (28.8–63.1)	46.4 (28.7–75.1)	−0.704	0.482
WCB/(10^9^/L)	11.8 (10.5–14.5)	12.7 (11.4–16.6)	−1.642	0.101
IL-1β/pg/mL	10.1 (5.3–15.9)	10.5 (5.3–16.5)	−0.291	0.771
IL-2/pg/mL	2.2 (2.0–2.6)	2.5 (1.9–4.2)	−1.855	0.064
IL-6/pg/mL	13.9 (7.6–42.5)	9.4 (4.3–19.1)	−1.578	0.115
TNF-α/pg/mL	1.8 (1.0–8.7)	1.4 (1.1–2.1)	−1.272	0.203
IFN-γ/pg/mL	6.5 (2.5–10.3)	4.1 (1.8–8.8)	−1.016	0.31
*Third day after surgery*				
CRP/(mg/L)	26.2 (11.1–67.1)	50.8 (23.6–72.3)	−2.452	0.014*
WCB/(10^9^/L)	7.7 (6.4–9.8)	7.5 (5.7–9.5)	−0.668	0.504
IL-1β/pg/mL	6.0 (1.9–8.7)	8.7 (3.5–14.6)	−2.434	0.015*
IL-2/pg/mL	1.6 (0.8–2.3)	1.6 (0.9–2.4)	−0.764	0.445
IL-6/pg/mL	10.0 (4.9–16.1)	4.5 (3.1–12.9)	−2.459	0.014*
TNF-α/pg/mL	1.7 (0.7–3.6)	1.3 (0.9–1.8)	−1.222	0.222
IFN-γ/pg/mL	6.6 (3.0–10.4)	4.3 (2.6–8.9)	−0.863	0.388

Compared with the SEA group, * *P* < 0.05. Data are presented in the form of median and interquartile range (M(IQR))

### Comparison of Incidence of Postoperative Adverse Events

There was no statistically significant difference in the incidence of postoperative adverse events between the two groups (*P* > 0.05), as presented in Table 5. Under the Clavien–Dindo (CD) grading system, both groups did not experience grade III or higher complications and neither group had serious complications such as anastomotic leakage, duodenal stump fistula, or cardiovascular disease. Compared with the SEA group, the EA group had no incidence of grade II or higher complications, but the difference was not statistically significant (*P* > 0.05), as presented in Table 5.

**Table 5:** Comparison of incidence of postoperative adverse events

Complication	EA (*n* = 50)	SEA (*n* = 48)	*χ*^2^-value	*P*-value
Yes	3 (6%)	4 (8.3%)	2.674	0.102
No	47 (94%)	41 (91.7%)		
*Clavien–Dindo classification*				
*n* (%)				
I	3 (100%)	1 (25%)	0.143	0.114
II	0 (0%)	3 (75%)		
III	0 (0%)	0 (0%)		

Data are presented in the form of percentage (%)

## Discussion

This study found that, compared with the sham EA group, the EA group exhibited a significant advancement in the first flatus time and bowel sound recovery time after surgery, which is consistent with results of similar research. Meanwhile, it was also suggested that EA could also shorten hospitalization duration and thereby improve the bed utilization rate. When comparing the inflammatory markers, it was observed that the levels of CRP and IL-1*β* in the EA group were significantly lower than those in the SEA group, with statistically significant differences. This suggests that EA effectively reduces the expression of inflammatory factors and alleviates the inflammatory response. However, the level of IL-6 was higher in the EA group, which differed from common acceptance. That may be associated with the relatively small sample size. The HRV analyses revealed that the values of SDANN, PNN50, and HF in the EA group were higher than those in the SEA group, indicating an obvious increase in vagus nerve tension and excitation following EA treatment. The decrease of inflammatory markers, CRP, and IL-1*β* was accompanied by the excitation of the vagus nerve, as detected by HRV indicators, which was probably related to the activation of the antiinflammatory pathway through the stimulation of the vagus nerve.

At present, it is general consensus that antiinflammation is the core of preventing and treating POI. After surgical trauma, stress response is prone to activate numerous inflammatory factors, leading to intestinal edema and weakened gastrointestinal motility. In this study, postoperative elevation in CRP, WCB, IL-1 *β*, IL-2, IL-6, TNF- *α*, IFN-*γ*, etc. indicated the occurrence of an inflammatory reaction after surgery. Lowering the levels of the inflammatory factors is beneficial for inhibiting intestinal inflammatory edema and promoting gastrointestinal function. There are two widely known pathways through which acupuncture stimulates the vagus nerve to exert immune and antiinflammatory effects. The first is the vagal–adrenergic axis. The vagus nerve afferent fibers drive the adrenal gland to release catecholamine antiinflammatory substances, reducing the production of cytokines TNF-*α* and IL-6 and thereby exerting systemic antiinflammatory effects.^13^ The second is the cholinergic antiinflammatory pathway (CAP). The α7 nicotinic acetylcholine receptors (α7nAchRs) on nearby macrophages are indirectly affected by vagus nerve efferent fibers, which prevent the macrophages from releasing proinflammatory cytokines, including TNF-α and IL-1*β*.^14^ It is no doubt that whichever pathway is activated by EA, the tension of vagus nerve has been demonstrated to rise.^13,14^

HRV is a recognized digital biomarker for assessing autonomic nervous tension.^15^ By recording and analyzing the small differences in the heartbeat intervals, the tension of the sympathetic and parasympathetic nervous systems can be digitized, allowing for quantitative assessment of autonomic function and its balance. Among the various indicators of HRV, SDANN is correlated with the overall function of the autonomic nervous system; PNN50 and HF reflect vagus nerve function; and LF is influenced by both sympathetic and parasympathetic nerves, reflecting the balance of the autonomic nervous system.^10,11^ With the remarkable progress of information technology and artificial intelligence, the era of digital healthcare has arrived.^16^ This study innovatively utilized a wearable dynamic electrocardiogram recorder and a continuous bowel sound auscultation recorder, which offered merits of noninvasive and dynamic monitoring, providing intelligent and visualized data for clinical diagnosis and treatment. The regulation of autonomic nervous system follows a clear circadian rhythm,^17,18^ which requires long-term and real-time monitoring. Existing HRV monitoring research focused on observing short-term frequency domain indicators^19–21^ and research on the temporal and frequency domain changes of 24 h or longer detection are lacking. The long-term wearable HRV monitoring device used in this study quantitatively reflected the vagal nerve tension and the balance between the vagal and sympathetic nerves through dynamic and visual measurements, providing real-time feedback on the neural regulation over a long period of time, thereby better evaluating the long-term effects of EA.

Existing methods for evaluating gastrointestinal function recovery mainly rely on the patients’ first flatus time, which is relatively subjective compared with the objective data from a recorder. However, the number of objective indicators is limited. The continuous bowel sound auscultation recorder assists in providing a more objective evaluation for postoperative intestinal dysfunction. In clinical practice, it guides patients to be fed without relying on subjective flatus time. Through using the continuous bowel sound auscultation recorder, the real-time gastrointestinal condition of postoperative patients can be detected, and adaptive adjustments can be made promptly, which also facilitates the implementation of personalized treatment. However, shortcomings are as follows: the sample size was small and gastrointestinal hormone indicators were not taken into consideration. In future studies, sample size should be increased, and more relevant indicators should be analyzed.

## Conclusions

The application of EA in postoperative patients with gastric cancer can shorten the recovery time of gastrointestinal function safely and effectively. It can also shorten the length of hospitalization. This study applied artificial intelligence devices to provide new directions for long-term perioperative medical monitoring and treatment.
